# Evaluation of Retinal Displacement After Rhegmatogenous Retinal Detachment Surgery: A Retrospective Single-Institution Study

**DOI:** 10.3390/medicina62020308

**Published:** 2026-02-02

**Authors:** Fabrizio Giansanti, Cristina Nicolosi, Diego Luciani, Giulio Vicini

**Affiliations:** 1 Eye Clinic, Neuromuscular and Sense Organs Department, Careggi University Hospital, 50134 Florence, Italy; fabrizio.giansanti@unifi.it (F.G.);; 2 Department of Neurosciences, Psychology, Drug Research and Child Health, University of Florence, 50121 Florence, Italy; 3 Ophthalmology Unit, Department of Medicine, Surgery and Neuroscience, University of Siena, 53100 Siena, Italy; diegoluciani8@gmail.com

**Keywords:** retinal displacement, retinal detachment, vitreoretinal surgery, vitrectomy

## Abstract

*Background and Objectives*: To evaluate the occurrence of retinal displacement using blue-fundus autofluorescence (BFAF) imaging in eyes treated for primary rhegmatogenous retinal detachment (RRD) and its associations with clinical factors, including macular status, detachment extent, baseline visual acuity, high myopia, postoperative visual recovery, and metamorphopsia. *Materials and Methods*: This retrospective observational study included 98 patients who underwent surgery for primary RRD at a single center. Surgical approaches included pars plana vitrectomy (PPV), phacovitrectomy, or scleral buckling, with tamponade agents such as SF_6_ gas (20%), silicone oil (≈1300 cSt), or air. Postoperative BFAF imaging assessed retinal displacement. Demographic and clinical data were recorded. *Results*: Macula-off detachments occurred in 56.1% of cases, while 43.9% were macula-on detachments. Phacovitrectomy was performed in 41.8%, simple vitrectomy in 33.7%, and scleral buckling in 24.5%. SF_6_ gas was the most used tamponade, while silicone oil was used in 13.3%. Retinal displacement was detected in 16.3% of cases, predominantly downward (81.25%) and less commonly upward (18.75%). Macula-off detachments were significantly associated with displacement (81.2% vs. 51.2%, *p* = 0.027). No significant associations were found with other parameters. Metamorphopsia was reported in 12.5% of patients with displacement and 4.9% without, though the difference was not statistically significant. *Conclusions*: Retinal displacement can occur after primary RRD repair, irrespective of tamponade, though it tended to be less frequent with silicone oil and in macula-on detachments. It is significantly more common in macula-off cases, even with immediate postoperative prone positioning. These findings emphasize the need to refine postoperative positioning protocols to reduce displacement and its sequelae. Further studies should explore the impact of retinal displacement on visual function, particularly metamorphopsia, in patients with preserved best-corrected visual acuity.

## 1. Introduction

Rhegmatogenous retinal detachment (RRD) is a severe and sight-threatening condition that occurs when a full-thickness retinal break allows liquefied vitreous to enter the subretinal space, separating the neurosensory retina from the underlying retinal pigment epithelium (RPE). This separation disrupts the essential metabolic and structural support provided by the RPE, leading to progressive photoreceptor dysfunction and vision loss if left untreated. RRD is considered an ocular emergency, requiring timely surgical intervention to reattach the retina and preserve vision. Advances in surgical instrumentation, techniques, and the use of intraocular tamponades like gas or silicone oil have significantly improved the anatomical success rates of these procedures [1]. However, despite achieving high reattachment rates, several postoperative complications may still occur, including one that has gained increasing attention in recent years: the retinal displacement. Retinal displacement refers to the unintended positional shift in the retina following RRD surgery, occurring when the retina reattaches but not in its original anatomical position [2,3]. First described in 2010 by Shiragami et al. [2], this phenomenon has been observed in a substantial proportion of cases—over 60% of eyes treated with PPV and gas tamponade in their study. Subsequent investigations have confirmed its occurrence not only after PPV but also after episcleral surgery [4,5,6]. While the anatomical reattachment of the retina may appear complete at the end of surgery, small amounts of subretinal fluid frequently persist. During the postoperative period, gravitational forces acting on this residual fluid can cause the retina to shift, particularly when patients are instructed to maintain specific positioning to promote tamponade effectiveness. Retinal displacement represents a unique challenge in the management of RRD. While its exact pathophysiology and clinical consequences are not fully understood, it is evident that this phenomenon is both common and multifactorial, influenced by surgical technique, tamponade choice, and patient-specific factors [3,7]. A better understanding of retinal displacement will not only aid in refining surgical approaches but also improve postoperative care and patient counseling, ultimately enhancing both anatomical and functional outcomes for individuals undergoing RRD surgery. Fundus autofluorescence (FAF) imaging has emerged as a key diagnostic tool for detecting retinal displacement [2,8,9]. By visualizing hyperautofluorescent lines adjacent to retinal blood vessels, FAF allows clinicians to identify shifts in the preoperative position of the retina.

The aim of this study was to evaluate, using BFAF imaging, the occurrence of retinal displacement in eyes treated for RRD and to investigate its potential correlations with preoperative and postoperative factors.

## 2. Material and Methods

This retrospective observational study included patients successfully treated for primary RRD at a single referral center (Careggi University Hospital, Florence, Italy) between 1 May 2023, and 30 June 2024. We included patients treated with PPV, phacovitrectomy, or scleral buckling, using silicone oil (≈1300 cSt) or gas (sulfur hexafluoride [SF_6_] 20%) as tamponade agents. The study adhered to the tenets of the Declaration of Helsinki and was approved by the Institutional Review Board. Informed consent was obtained from all patients included in the study. Patients with the following clinical and anatomical factors known to cause retinal slippage were deliberately excluded: previous macular surgery, pre-existing macular epiretinal membrane (ERM), macular proliferative vitreoretinopathy (PVR), giant retinal tear, and pre-existing grade C or superior PVR [10]. Other exclusion criteria included: age < 18 years, any retinal disease such as age-related maculopathy, diabetic retinopathy, retinal vein or artery occlusion; any optic nerve disease such as glaucoma or optic neuropathy; previous vitreoretinal surgery; recurrent RRD; combined rhegmatogenous and tractional RD; media opacities obscuring adequate clinical evaluation and imaging (e.g., poor-quality fundus autofluorescence [FAF] images); significant systemic diseases (diabetes or other systemic pathologies that may affect the retinal microstructures) or cognitive impairment that precluded adequate clinical and instrumental evaluation. Patients were included only if high-quality blue-fundus autofluorescence (BFAF) imaging, performed at least one month after surgery, was available to assess the presence of postoperative macular displacement.

### 2.1. Surgical Procedure

All patients underwent the same standardized surgical procedures (PPV, phacovitrectomy, or scleral buckling) performed under local retrobulbar or general anesthesia by a single experienced vitreoretinal surgeon (F.G.). Three-port 25- or 23-gauge PPV were performed in all cases with a CONSTELLATION Vision System (Alcon Laboratories, Fort Worth, TX, USA). Vitrectomy procedures were performed using an OPMI LUMERA 700 surgical microscope (Carl Zeiss Meditec, LUMERA 700 surgical microscope (Carl Zeiss Meditec, Jena, Germany) and a non-contact wide-angle RESIGHT viewing system (Zeiss, Oberkochen, Germany). Patients over the age of 50 underwent combined cataract surgery (phacovitrectomy). If required, a posterior vitreous detachment was created using suction with the vitrectomy probe around the optic nerve head. Central and peripheral vitrectomy with scleral indentation was performed, ensuring thorough shaving of the vitreous base to alleviate vitreous traction on retinal tears. Perfluorocarbon liquid (PFCL) was employed to flatten the retina in all cases. Following a meticulous 360° inspection of the retinal periphery, a fluid/air exchange was performed, with a focus on maximizing subretinal fluid drainage through the original retinal breaks. No posterior retinotomies were carried out. Retinal breaks were treated with endolaser photocoagulation. A fluid–air exchange, followed by either air–gas or air–silicone oil (SO) exchange, was performed in all cases. Sulfur hexafluoride gas (SF_6_, 20%) or silicone oil (≈1300 cSt; polydimethylsiloxane [PDMS]) was used as the internal tamponade. Specifically, SF_6_ was selected for cases without proliferative vitreoretinopathy (PVR) or with PVR grade A, while silicone oil was used for cases presenting with PVR grade B. After surgery, patients immediately assumed a strict face-down position in the operating room and were instructed to maintain this posture for approximately 48 h to optimize retinal reattachment and postoperative recovery. This postoperative positioning protocol was applied to all vitrectomy and phacovitrectomy cases.

In patients who underwent scleral buckling, an encircling band was combined with either a radial or segmental buckle, depending on the surgeon’s clinical judgment and the specific characteristics of the retinal detachment. The encircling band provided 360° support to the globe, alleviating vitreoretinal traction and securing the retinal breaks. Radial buckles were used in cases where localized retinal tears required precise indentation, while segmental buckles were applied to support broader areas of retinal pathology. The surgical technique involved careful suturing of the buckle material to the sclera to ensure proper placement and sufficient pressure for retinal reattachment. Subretinal fluid drainage was selectively performed based on the extent of fluid accumulation. In all cases, air was used as part of the procedure to facilitate reattachment and support of the retina during and after surgery. For scleral buckling procedures, postoperative instructions emphasized maintaining a face-down position for the first 24 h, oriented according to the location of the treated break and the surgeon’s recommendation.

### 2.2. Data Collection and Analysis

The medical records of patients treated for primary RRD who met the inclusion criteria were collected and analyzed. Before the surgery, all the patients included in the study underwent a comprehensive ophthalmic examination including best-corrected visual acuity (BCVA) measurement using the Snellen visual acuity chart, anterior segment examination, dilated fundoscopy and macular spectral domain optical coherence tomography (SD-OCT). Clinical and demographic characteristics of the patients were recorded, including macular involvement status, presence of high myopia, affected retinal quadrants, location and number of retinal breaks, presence of PVR, and lens status at baseline. The final number of retinal breaks was determined based on intraoperative findings following complete vitreous base shaving and 360° peripheral inspection. Eyes with no retinal breaks detected preoperatively but with one break identified intraoperatively were classified as single-break cases. If additional retinal breaks were found during surgery, they were documented and included in the final data analysis. We collected postoperative data, including BCVA at 1 month and 6 months, type of surgery, type of tamponade agent used (air, gas, silicone oil), and the presence of metamorphopsia, which was subjectively reported by patients and confirmed using the Amsler grid.

Blue-fundus autofluorescence images (B-BAF) and macular SD-OCT scans were acquired after pupillary dilation with topical tropicamide with Spectralis HRA + OCT (Heidelberg Engineering, Heidelberg, Germany), which combines a confocal scanning laser ophthalmoscope with spectral-domain OCT. The presence of macular displacement on BFAF images was assessed by identifying retinal vessel printings (RVPs) and their direction (upward or downward) relative to the adjacent retinal vessels. RVPs were defined as hyperautofluorescent lines running approximately parallel to adjacent retinal vessels, separated from them, and closely matching their caliber and orientation. The detection of RVPs was considered evidence of retinal displacement.

### 2.3. Statistical Analysis

Statistical analysis was performed using SPSS Statistics (SPSS Inc., Chicago, IL, USA) software for Mac (Version 26.0). Continuous variables were presented as a mean ± SD and categorical variables as number and percentages. Preoperative and postoperative clinical characteristics according to the presence or absence of retinal displacement were compared using a two-tailed Student’s *t*-test or Chi-square test with 95% confidence intervals. The chosen level of statistical significance was *p*-value < 0.05.

## 3. Results

### 3.1. Demographic and Baseline Clinical Characteristics

The study included 98 eyes from 98 patients with primary RRD successfully treated with surgery. Of these patients, 54 (55.1%) were male and 44 (44.9%) were female, with a mean age of 63.6 ± 12.3 years. Preoperative decimal best-corrected visual acuity (BCVA) was 0.36 ± 0.31, ranging from hand motion (decimal 0.005 according to Schulze-Bonsel et al., [11]) to 1.0. In 51 (52%) patients, the right eye was involved, while in 47 (48%) patients, the left eye was affected. At baseline, 65 patients (66.3%) were phakic, and 33 (33.7%) were pseudophakic. Forty-three patients (43.9%) had high myopia, defined as ≥6 diopters.

Regarding preoperative clinical data, 55 patients (56.1%) had macula-off detachments, while 43 patients (43.9%) had macula-on detachments. In the majority of cases (*n* = 51, 52%), the detachment involved both the superior and inferior retinal quadrants. In 37 cases (37.8%), only the superior quadrants were involved, while in 10 cases (10.8%), only the inferior quadrants were affected. Preoperatively, a single retinal tear was identified in 69 (70.4%) cases, two tears in 22 cases (22.5%), and more than two tears in 7 cases (7.1%). A PVR of grade A or B was present in 8 cases (8.2%), while cases with more advanced PVR were excluded (see Section 2). Demographic and clinical characteristics of the patients included in the study are reported in Table 1.

### 3.2. Postoperative Data and Macular Displacement

**Figure 1:**
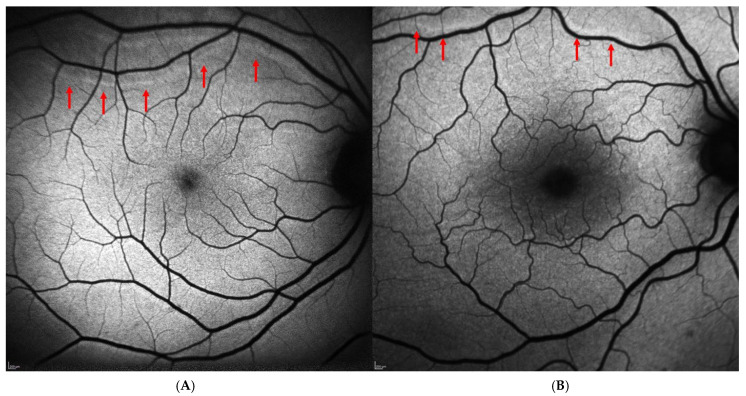
Postoperative retinal displacement on blue-fundus autofluorescence images (Spectralis HRA + OCT, Heidelberg Engineering, Heidelberg, Germany). (**A**) Upward retinal displacement following scleral buckling surgery for macula-on rhegmatogenous retinal detachment. (**B**) Downward retinal displacement in a patient with macula-off retinal detachment who underwent PPV with SF_6_ 20% tamponade. In both cases, retinal vessel printings (red arrows) are visible as hyperautofluorescent lines running approximately parallel to the adjacent retinal vessels, clearly separated from them, and closely mirroring their caliber and orientation.

Regarding the postoperative data, 41 patients (41.8%) underwent phacovitrectomy, 33 patients (33.7%) underwent simple vitrectomy, and 24 patients (24.5%) underwent scleral buckling. The most frequently used tamponade was 20% SF_6_ gas, while silicone oil (≈1300 cSt) was used in 13 cases (13.3%), and air was used exclusively in the scleral buckling cases (24 cases, 24.5%). The postoperative decimal BCVA was 0.49 ± 0.24 at one month and 0.52 ± 0.24 at six months, indicating a slight trend of improvement over time. Metamorphopsia was reported subjectively by 6 of the 98 patients (6.1%) and confirmed using the Amsler grid. No patients in the study cohort reported postoperative diplopia. Retinal displacement was identified on BFAF images in 16 out of 98 cases (16.3%). In most cases, it was downward (13 out 16 cases, 81.25%), while upward displacement was observed in 3 cases (18.75%). Figure 1 shows an example of “upward” (Figure 1A) and “downward” (Figure 1B) displacement in two patients included in the study. Postoperative data of the patients included in the study are reported in Table 2.

## 4. Data Analysis

We evaluated the existence of statistically significant differences between demographic and clinical parameters at baseline and postoperatively in patients with retinal displacement (*N* = 16) compared to those without retinal displacement (*N* = 82). The analysis revealed a statistically significant difference only for the presence of macula-off and macula-on detachments, with a higher percentage of patients exhibiting macula-off detachment in the retinal displacement group compared to the group without retinal displacement (81.2% vs. 51.2%, *p* = 0.027). No statistically significant differences were observed for the other evaluated parameters. Regarding tamponade types, the use of gas tamponade was more frequent in the group with retinal displacement compared to the group without displacement (75% vs. 59.8%), while the use of silicone oil and air was more common in the group without displacement (12.5% vs. 13.4% and 12.5% vs. 26.8%, respectively). However, these differences did not reach statistical significance between the two groups (*p* = 0.442). We performed a subgroup analysis including only patients who underwent vitrectomy (*N* = 74) to highlight differences between gas and silicone oil tamponade. Even in this case, the result was statistically non-significant (*p* = 0.790). Retinal displacement was more frequent in the vitrectomy group (87.5%) compared to the episcleral surgery group (12.5%), though no statistically significant difference was observed when compared to the group without displacement. Preoperative BCVA did not differ significantly between the groups (*p* = 0.262). Postoperative outcomes showed improved BCVA in both groups, with slight improvements over time, from one to six months, similar for patients with and without retinal displacement (*p* > 0.05). Notably, metamorphopsia was reported by 2 out of 16 patients (12.5%) with retinal displacement, while it was observed in 4 out of 82 patients (4.9%) without retinal displacement. However, this was not significantly correlated with the presence of retinal displacement (*p* = 0.245). The complete data analysis between the groups is presented in Table 3.

## 5. Discussion

RRD remains one of the leading causes of vision loss that requires urgent ophthalmic intervention. Over the past few decades, advancements in surgical techniques have significantly improved the success rates of retinal reattachment. However, despite these achievements in anatomical reattachment, many patients continue to experience suboptimal functional outcomes. Common complaints include visual distortions such as metamorphopsia and/or anisometropia, which can significantly affect quality of life. In some cases, these residual visual disturbances may be linked to retinal displacement, a phenomenon that has gained increasing attention in recent years [3,12].

The concept of retinal displacement following RRD repair was first introduced in 2010 by Shiragami and colleagues, when hyperautofluorescent lines were detected using fundus autofluorescence (FAF) imaging [2]. These lines, referred to as “retinal vessel printings” (RVPs) or “retinal pigment epithelial vessel ghost lines,” are thought to represent the original position of retinal vessels before detachment occurred. The presence of RVPs is considered indicative of retinal displacement in patients who have undergone RRD repair. Shiragami and colleagues observed retinal displacement in 62.8% of patients, linking it to increased metabolic activity in the retinal pigment epithelium (RPE), which was previously shielded by the retinal vessels and then acutely exposed to light postoperatively due to the displacement of the vessels from their original positions.

Subsequent research has demonstrated that retinal displacement is not an uncommon occurrence and may correlate with poorer functional outcomes [3,13,14]. The presence of RVPs can serve as a visual marker for displaced retinal tissue and may provide valuable insights into the underlying reasons for diminished postoperative visual function. Long-term effects of retinal displacement remain underexplored, but studies suggest that it may be linked to persistent visual disturbances, such as metamorphopsia, which significantly impair patients’ ability to perform daily tasks. Furthermore, the psychological burden associated with these distortions often impacts the quality of life, underscoring the importance of addressing this issue in future research.

Various factors have been proposed to contribute to retinal displacement, including the surgical techniques used, the extent of retinal tears, and the type of tamponade agents employed during the procedure. While the exact mechanisms of retinal displacement remain under investigation, it is clear that this phenomenon is a significant consideration when evaluating the functional success of RRD repair. Further investigation into the risk factors and long-term consequences of retinal displacement is essential in order to optimize postoperative management and improve visual outcomes for patients undergoing RRD surgery.

In our study, we analyzed data from 98 patients who successfully underwent retinal detachment surgery using vitrectomy and scleral buckling surgery, and were evaluated postoperatively with blue autofluorescence imaging. Retinal displacement was observed in 16 out 98 patients (16.3%). Literature reports varying incidence rates, influenced by factors such as inclusion criteria, surgical techniques, tamponade selection, and postoperative management strategies [3,7]. Retinal displacement, defined as the postoperative misalignment of retinal photoreceptor cells detectable on fundus autofluorescence, ranges from 4.5% to 62.8% following RRD surgery.

The frequency of displacement observed in our cohort aligns with those reported in other studies, although it is lower than expected. Given the preoperative characteristics and existing literature, we expected a higher overall rate of displacement.

The frequency of displacement observed in our cohort aligns with those reported in other studies, although it is lower than expected compared to the 62.8% incidence described by Shiragami et al. in the first report of postoperative retinal displacement following PPV with gas tamponade and the up-to-60% rate summarized in the recent systematic review by Mason et al. [2,3]. Based on the preoperative characteristics of our cohort and previous reports, a higher overall rate of displacement would have been expected.

We believe this result can be attributed to the exclusion of patients with potential confounding factors, such as PVR grade C or worse, giant retinal tears, ERM, and patients who had undergone previous macular surgery, all of which are known risk factors for retinal displacement. We believe that the strict exclusion criteria, based on existing evidence, contributed to the high quality of our sample. Additionally, all patients treated at our center and included in this study were advised to maintain a strict face-down posture for the first two hours post-surgery and were instructed to follow these guidelines for the subsequent 24–48 h, depending on the type of surgery. Recent studies suggest that face-down positioning after surgery may reduce complications, such as postoperative retinal displacement and outer retinal folds, though further high-quality data is needed [15].

The use of tamponades, whether gas or silicone oil (SO), has been linked to unintentional retinal displacement. Shiragami and colleagues first reported retinal displacement in a cohort of 43 patients treated with PPV and gas tamponade for bullous and superior RRDs, with 62.8% (27/43) of patients experiencing displacement [2]. However, no immediate prone positioning was recommended; instead, patients were advised to sit. The authors hypothesized that in gas-filled eyes, sitting might encourage the downward shift in residual subretinal fluid, causing displacement due to gravity. Dell’Omo and colleagues observed lower displacement rates of 35% (7/20) and 28.6% (16/56) when immediate postoperative face-down positioning was employed [14,16]. Conversely, Cobos and colleagues recorded retinal displacement in 60% (12/20) despite face-down positioning [13]. Chelazzi and colleagues found a retinal displacement rate of 28.2% (11/39) in macula-off cases and 0% (0/32) in macula-on cases, when patients were placed supine after surgery for macula-off RRD or when complete subretinal fluid drainage had occurred [17]. Kumar and colleagues recommended prone or lateral positioning depending on the location of the primary retinal break, with a retinal displacement rate of 6.4% (5/78) [18]. In our cohort, the use of gas tamponade was more frequent in the group with retinal displacement compared to those without (75% vs. 59.8%); however, no statistically significant difference was observed between the two groups in tamponade use.

We did not find any association between the extent of RRD and the incidence of retinal displacement. In our population, factors such as the number of retinal tears, the quadrants involved, and the presence of PVR (grades A–B) did not influence the proportion of patients with retinal displacement. These findings are consistent with some previous studies but differ from others, which may be attributed to differences in sample composition or study methodologies [3,7].

In our cohort, we observed a statistically significant difference regarding preoperative macula status (macula-off vs. macula-on detachments), with a higher percentage of patients in the retinal displacement group showing macula-off detachment compared to those without retinal displacement (81.2% vs. 51.2%, *p* = 0.027). Retinal displacement appears to be more prevalent in macula-off detachments, even with immediate postoperative prone positioning protocols. Shiragami et al. found that macula-off RRD was associated with a higher risk of retinal displacement, with a statistically significant difference (*p* = 0.016) [2]. Similarly, Chelazzi et al. reported that among 71 patients, 28.2% experienced retinal slippage, all of whom had macula detachment, while none of the 32 macula-on patients experienced retinal displacement [17].

Based on the findings from our study and recent evidence in the literature, we emphasize the crucial importance of adopting and maintaining a face-down position promptly, particularly for patients in high-risk categories such as those with gas tamponades or macula-off RRD [3,7]. There is growing evidence supporting the hypothesis that retinal displacement may occur as a result of subretinal fluid movement beneath the delicate, elastic retina, driven by the buoyant force of the tamponade [19,20]. This force stretches the retina, which is consistent with the observed downward displacement of the retina, as subretinal fluid tends to move inferiorly due to gravity and head positioning [3]. In our cohort, the majority of displacements were downward, aligning with this hypothesis (13 out of 16 cases, 81.25%). This hypothesis may also explain why immediate face-down positioning, without a preceding head-elevated position, results in less retinal displacement compared to the ‘position-to-the-break’ technique, in which postoperative head positioning is oriented toward the retinal break rather than strictly face-down [21]. Alternative postoperative posturing strategies, including sequential face-up followed by face-down positioning, have also been proposed to promote uniform subretinal fluid redistribution and facilitate photoreceptor realignment [22,23]. Although such dual protocols may influence retinal reattachment dynamics, all patients in our study followed an immediate and continuous face-down regimen, ensuring standardization across the cohort. The immediate face-down positioning facilitates the flow of posterior subretinal fluid in all directions, as opposed to a solely inferior flow, potentially contributing to the reduced incidence of inferior retinal displacement. Additionally, the hypothesis is consistent with the observation of lower rates of retinal displacement in procedures such as PPV with silicone oil, pneumatic retinopexy, and scleral buckling. Both SO and the small expansile gas bubble, which can be employed in episcleral surgery, exert less buoyant force on the retina compared to a full gas fill, as their contact area and pressure are reduced.

PFCL, when used as a temporary intraoperative tamponade, is thought to effectively stabilize the retina during surgery, particularly in macula-off detachments where precise alignment is crucial. It helps displace subretinal fluid anteriorly, facilitating more complete drainage and potentially reducing the need for posterior drainage retinotomy [24]. PFCL may also contribute to improved postoperative retinal positioning, potentially leading to better visual outcomes. However, the relationship between PFCL use and unintentional retinal displacement remains controversial. Some studies have suggested that PFCL may reduce postoperative displacement by promoting rapid subretinal fluid removal and improving retinal apposition [22,23,25], while others have proposed that transient mechanical stress or micro-movements induced during PFCL manipulation might contribute to subtle retinal layer misalignment [7]. One study described PFCL as a temporary stabilizing agent that enhances intraoperative retinal stability, although this conclusion was not supported by specific statistical data [24]. Conversely, two studies reported significantly smaller postoperative macular shifts with PFCL use [22,25], whereas dell’Omo et al. found no significant effect on retinal displacement, despite its use in 80% of cases [26]. In our study, PFCL was systematically used in all patients who underwent PPV or phacovitrectomy, with fluid–air exchange preceding both gas and silicone oil tamponades. This uniform intraoperative use ensured consistent surgical conditions but limited the ability to analyze PFCL as an independent factor influencing postoperative displacement. Therefore, any potential protective or risk-increasing effect of PFCL could not be distinguished analytically, representing an inherent limitation of our dataset that should be considered when interpreting the results.

Regarding functional outcomes, studies in the literature suggest that retinal displacement did not affect postoperative BCVA. In our cohort, we also did not observe any statistically significant differences in postoperative BCVA at 1 and 6 months between patients with and without retinal displacement. Several studies have investigated metamorphopsia in patients following RRD repair [3,7]. Four of these studies reported significant subjective visual distortion associated with retinal displacement, with incidence ranging from 83% to 24%, and nearly all affected patients presented with macula-off detachments [17,21,22,26]. It is important to note that macula-off detachments can lead to various retinal changes (e.g., irregularities in the outer retinal bands, reduced retinal thickness, outer retinal folds, CME, or subretinal fluid), all of which could contribute to postoperative metamorphopsia. Isolating the specific contribution of each factor to metamorphopsia remains challenging and may not be feasible. In our cohort, metamorphopsia was reported by 2 out of 16 patients (12.5%) with retinal displacement, while it was observed in 4 out of 82 patients (4.9%) without retinal displacement. However, despite being more frequent in the displacement group, no statistically significant difference was observed. It is worth noting that in these patients reporting subjective distortion, significant structural changes in OCT were excluded at the time of evaluation.

We acknowledge several limitations in our study, including its retrospective design and the relatively small number of patients with documented retinal displacement. However, our analysis involved a large and well-characterized cohort that encompassed various standardized RRD repair procedures while rigorously excluding known confounding factors. The small number of eyes with displacement (*N* = 16) may have limited the statistical power of some subgroup analyses—particularly those comparing tamponade agents and evaluating the presence of metamorphopsia. Therefore, the absence of statistically significant associations should be interpreted with caution, as it may reflect either a true lack of effect or a type II error related to the limited sample size. In addition, the small number of displacement cases precluded a fully powered, procedure-specific analysis (e.g., scleral buckling versus PPV). Despite these constraints, our study provides valuable insights into retinal displacement and its potential contributing factors, offering a basis for future prospective and larger-scale investigations.

Quantitative assessment of retinal displacement amplitude (e.g., in degrees or micrometers) was not feasible due to the retrospective design and the lack of preoperative registration-based imaging. Only the direction of vessel displacement could be recorded. This methodological limitation should be addressed in future prospective studies employing standardized image registration software. Metamorphopsia was assessed qualitatively using the Amsler grid, as quantitative tools such as M-CHARTS or DPD tests were not available during the study period.

## 6. Conclusions

In conclusion, this study highlights that unintentional retinal displacement following primary RRD repair can occur with both gas and silicone oil tamponades, although its incidence is lower in cases involving silicone oil and macula-on detachments. Retinal displacement is more frequently observed in macula-off detachments, even with immediate postoperative prone positioning protocols. Our findings underscore the importance of appropriate postoperative positioning, not only to prevent well-established complications but also to reduce the risk of inadvertent retinal displacement.

The association between retinal displacement and metamorphopsia warrants further exploration, as high-quality evidence is needed to clarify its impact on visual function, even when BCVA remains unaffected. Addressing this relationship will be challenging, especially in macula-off detachments where numerous variables, such as retinal structural changes and subretinal fluid dynamics, influence outcomes.

Future research should focus on evaluating the potential protective role of PFCLs during surgery and heavy silicone oil as a tamponading agent in mitigating the risk of displacement. Additionally, the concept of “low-integrity reattachment”, defined as the reattachment of the retina in a slightly displaced or non-physiological configuration due to incomplete elimination of subretinal fluid or residual tractional forces, should be further examined, as greater awareness and understanding of this phenomenon among vitreoretinal surgeons may lead to refinements in surgical techniques for primary RRD repair. Comprehensive longitudinal studies with larger patient cohorts are essential to strengthen the evidence base, optimize both intraoperative and postoperative management strategies, and ultimately refine clinical practice guidelines to minimize retinal displacement and its functional implications.

## Figures and Tables

**Table 1:** Demographic and baseline clinical characteristics of patients included in the study.

Number of Patients (Eyes)	98 (98)
Age (years) at diagnosis	
Mean ± SD	63.6 ± 12.3
Median	63
Range	37–84
Gender, *n* (%)	
Male	54 (55.1%)
Female	44 (44.9%)
Preoperative BVCA (decimal)	
Mean ± SD	0.36 ± 0.31
Range	0.005 (hand motion)–1.0
Laterality, *n* (%)	
Right	51 (52.0%)
Left	47 (48.0%)
Lens status at baseline, *n* (%)	
Phakic	65 (66.3%)
Pseudophakic	33 (33.7%)
High Myopia (≥6 diopters), *n* (%)	
Yes	43 (43.9%)
No	55 (54.1%)
Macular involvement, *n* (%)	
Yes (macula-off)	55 (56.1%)
No (macula-on)	43 (43.9%)
Detachment location, *n* (%)	
Superior	37 (37.8%)
Inferior	10 (10.2%)
Superior + Inferior	51 (52%)
Retinal tears number/eye, *n* (%)	
1	69 (70.4%)
2	22 (22.5%)
>2	7 (7.1%)
Proliferative vitreoretinopathy, *n* (%)	
Yes	8 (8.2%)
No	90 (91.8%)

**Table 2:** Surgical and postoperative data of patients included in the study.

Number of Patients (Eyes)	98 (98)
Surgical procedure, *n* (%)	
Phacovitrectomy	41 (41.8%)
Vitrectomy	33 (33.7%)
Scleral buckling	24 (24.5%)
Tamponade agent, *n* (%)	
Gas (SF_6_ 20%)	61 (62.2%)
Silicone oil (≈1300 cSt)	13 (13.3%)
Air (scleral buckling only)	24 (24.5%)
Macular displacement, *n* (%)	
Yes	16 (16.3%)
No	82 (83.67%)
Metamorphopsia, *n* (%)	
Yes	6 (6.1%)
No	92 (93.9%)
Postoperative BVCA (decimal)	
1 month (mean ± SD)	0.49 ± 0.24
6 months (mean ± SD)	0.52 ± 0.24

**Table 3:** Preoperative and postoperative data by the presence or absence of retinal displacement.

	Macular Displacement		
	Yes (*N* = 16)	No (*N* = 82)	*p*-Value
Age (years) at diagnosis			0.456 ^a^
Mean ± SD	65.75 ± 14.9	63.22 ± 11.8	
Gender, *n* (%)			0.920 ^b^
Male	9 (56.3%)	45 (54.9%)	
Female	7 (43.7%)	37 (45.1%)	
Preoperative BVCA (decimal)			0.262 ^a^
Mean ± SD	0.28 ± 0.28	0.37 ± 0.31	
Laterality, *n* (%)			0.468 ^b^
Right	7 (43.7%)	44 (53.7%)	
Left	9 (56.3%)	38 (46.3%)	
Lens status at baseline, *n* (%)			0.167 ^b^
Phakic	13 (81.2%)	52 (63.4%)	
Pseudophakic	3 (18.8%)	30 (36.6%)	
High Myopia (≥6 diopters), *n* (%)			0.937 ^b^
Yes	7 (43.7%)	35 (42.7%)	
No	9 (56.3%)	47 (57.3%)	
Macular involvement, *n* (%)			0.027 ^b^
Yes (macula-off)	13 (81.2%)	42 (51.2%)	
No (macula-on)	3 (18.8%)	40 (48.8%)	
Detachment location, *n* (%)			0.203 ^b^
Superior	5 (31.3%)	32 (39%)	
Inferior	0 (0%)	10 (12.2%)	
Superior + Inferior	11 (68.8%)	40 (48.8%)	
Retinal tears number/eye, *n* (%)			0.122 ^b^
1	9 (56.3%)	60 (73.2%)	
2	4 (25%)	18 (21.9%)	
>2	3 (18.8%)	4 (4.9%)	
Proliferative vitreoretinopathy, *n* (%)			0.760 ^b^
Yes	1 (6.3%)	7 (8.5%)	
No	15 (93.7%)	75 (91.4%)	
Surgical procedure, *n* (%)			0.223 ^b^
Phacovitrectomy/Vitrectomy	14 (87.5%)	60 (73.2%)	
Scleral buckling	2 (12.5%)	22 (26.8%)	
Tamponade agent, *n* (%)			0.442 ^b(^*^)^
Gas (SF_6_ 20%)	12 (75%)	49 (59.8%)	0.790 ^b(^**^)^
Silicone oil (≈1300 cSt)	2 (12.5%)	11 (13.4%)	
Air (scleral buckling only)	2 (12.5%)	22 (26.8%)	
Metamorphopsia, *n* (%)			0.245 ^b^
Yes	2 (12.5%)	4 (4.9%)	
No	14 (87.5%)	78 (95.1%)	
Postoperative BVCA (decimal), *n* (%)			
1 month (mean ± SD)	0.46 ± 0.20	0.50 ± 0.25	0.553 ^a^
6 months (mean ± SD)	0.51 ± 0.21	0.52 ± 0.24	0.857 ^a^

^a^ Student’s *t*-test. ^b^ Chi-square test. * Comparison performed on the entire cohort (*N* = 98). ** Comparison of tamponade agents (gas vs. silicone oil) limited to patients who underwent PPV or phacovitrectomy (*N* = 74).

## Data Availability

The data presented in this study are available on request from the corresponding author. The data (original imaging) are not publicly available due to privacy issues.
